# Phase transitions of ionic fluids in nanoporous electrodes

**DOI:** 10.1140/epje/s10189-023-00350-2

**Published:** 2023-10-04

**Authors:** Ayeh Emrani, Clifford E. Woodward, Jan Forsman

**Affiliations:** 1 Theoretical Chemistry, Lund University, P.O. Box 124, 221 00 Lund, Sweden; 2 University College, University of New South Wales (ADFA), Canberra, ACT 2600 Australia

## Abstract

**Abstract:**

In this work, we utilise grand canonical Metropolis Monte Carlo simulations, to establish pore-induced freezing of restricted primitive model fluids. A planar pore model is utilised, with walls that are initially neutral, and either non-conducting or perfectly conducting. The phase of the confined electrolyte (solid/fluid) displays an oscillatory dependence on surface separation, in narrow pores. Conditions are chosen so that the bulk is composed of a stable fluid electrolyte. The tendency for the electrolyte to freeze in narrow pores is somewhat stronger in systems with non-conducting walls. We also demonstrate that an applied potential will, above a threshold value, melt a frozen electrolyte. In these cases, the capacitance, as measured by the average surface charge density divided by the applied potential, will be almost vanishing if the applied potential is below this threshold value. We do not see any evidence for a “superionic fluid”, which has been hypothesised to generate a strong capacitance in narrow pores, due to an efficient screening of like-charge repulsions by image charges.

**Graphic abstract:**

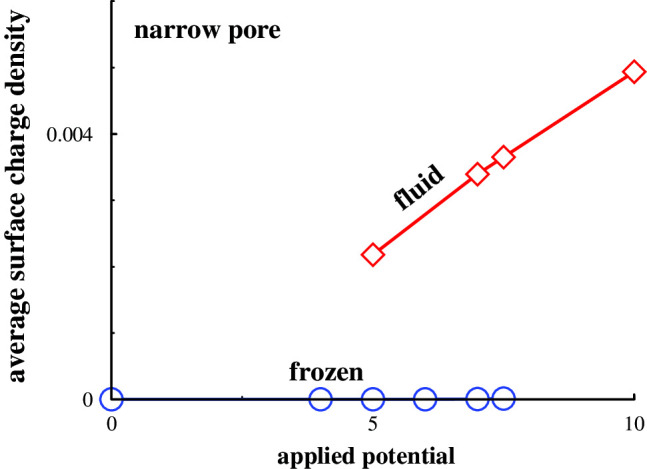

**Supplementary Information:**

The online version contains supplementary material available at 10.1140/epje/s10189-023-00350-2.

## Introduction

The behaviour of ionic fluids in confined spaces is of considerable scientific and industrial interest, especially considering the development of modern electric double layer capacitors, EDLCs, where nanoporous electrodes often are utilised. One of the reasons to construct electrodes in this way is that this will facilitate a large effective area of the capacitor.

Many “room temperature” ionic liquids, ILs, actually need a somewhat elevated temperature to melt, and even in other cases, the melting point is often not far below “room temperature”. Hence, freezing transitions that are affected by confinement [1–9] may have a substantial effect on the performance of EDLCs. A relevant aspect is of course the geometry of the confined space, as well as possible crystallinity of the confining surfaces, but even with a very simple geometry, and structureless confining walls, the conductivity of the material of which the surfaces are made, can play an important role [9–11]. Moreover, the application of a potential may itself generate phase transitions [7, 12], with a concomitant impact on electrochemical performance. For instance, Kondrat and Kornyshev [13–15] suggested that an electrolyte, or ionic liquid, confined between conducting walls may undergo a phase transition to a “superionic state” at high applied voltages. This is then accompanied by a very high, or even diverging, capacitance, brought about by an efficient screening of the repulsion between confined ions, most of which have the same charge.^1^ This suggestion was, at least in part, motivated by experimental data that indicated an anomalous increase of the capacitance in narrow pores. It should be noted, however, that these findings were subsequently disputed [16], and there appears to be no consensus on the matter.

Weingarth et al. [7] conducted differential scanning calorimetry measurements of ILs in the bulk, as well as in nanoporous carbon, at various temperatures. They not only found significant melting point depressions, but also that the melting temperature dropped further upon the application of a potential. Liu et al. used similar techniques to establish melting point depressions of various ILs on nano-$SiO_x$ particles [2]. A seeming contradiction to these results are data found by Comtet et al. [9], who instead noted freezing transitions of ILs upon confinement, using an atomic force microscope. The surface separation at which a freezing transition occurred, depended strongly on the conductivity of the substrate surface, i.e. on the surface onto which an IL droplet was applied. With a highly conducting substrate (Platinum) a freezing transition took place at astonishingly large separations - more than 100 nm. Switching substrate to mica resulted in a drop of the “freezing separation” by an order of magnitude.

In this work, we will perform Metropolis Monte Carlo simulations, using a very simplified model of a molten salt, or an ionic liquid, where all ions are spherically symmetric and have the same size. Moreover, the confining surfaces, that may be charged, are perfectly flat. Two different kinds of surfaces are considered: non-conducting (with the same dielectric constant as in the electrolyte), or perfectly conducting. Our focus is how melting/freezing transitions depend on pore size, applied voltage, and the nature of the confining surfaces. The qualitative behaviours that we will establish are in some respect similar to those by Kiyohara *et al.* [3], but the fluids they investigated were considerably more dilute than our systems (about 20 times), and the phase transitions they observed were akin to those between a liquid and a gas.

It is obvious that our model lacks several features that may be relevant in specific cases, such as ion size asymmetry, ion polarisability, surface structure, and so on. However, the model does afford the advantage of being composed of a minimal set of parameters, which should allow relatively straightforward interpretations. These will hopefully be useful for qualitative predictions, and permit scrutiny of some generic mechanisms that govern responses to changes of confinement, applied voltage, and surface conductivity.

## Model and methods

The ions are described as softly repulsive spheres, carrying a central elementary charge (i.e. a 1:1 salt). The Coulombic ion-ion interaction, $\Phi _C$, is given by:

1 $$\begin{aligned} \beta \Phi _C(r_{ij}) = \frac{Z_i Z_j l_B}{r_{ij}} \end{aligned}$$

where $r_{ij}$ is the mutual distance between ions *i* and *j*, whereas $Z_i$ is the valency of ion *i*. The inverse thermal energy is denoted by $\beta$, and the Bjerrum length, $l_B$ is given by $l_B = \beta e^2/(4\pi \epsilon _r\epsilon _0)$, where *e* denotes the elementary charge, and $\epsilon _0$ is the permittivity of vacuum. We assume a fluid dielectric constant $\epsilon _r = 5$, and the temperature is set to 300 K. This results in a Bjerrum length of about 111 Å. The soft repulsion, $u_r$, acting between two ions separated by *r* has the form,

2 $$\begin{aligned} \beta u_r(r) = \left( \frac{d}{r}\right) ^{12} \end{aligned}$$

where $$d = 4$$ Å.

The simulated system is composed of an electrolyte between two flat surfaces with infinite extension in the (*x*, *y*) plane. These surfaces are located at $z=-h/2$, and $z=h/2$. A hard wall repulsion ensures that the *z*-coordinate of the centre of every ion is confined so that, $(d-h)/2 \le z \le (h-d)/2$. The simulation box is also limited along the (*x*, *y*) directions, with $|x|\le L/2$ and $|y|\le L/2$. Periodic boundary conditions are applied along the (*x*, *y*) directions, in order to mimic macroscopic surfaces. Each surface furthermore defines a plane of dielectric discontinuity. We will consider two different scenarios: walls with the same dielectric constant as in the confined electrolyte, or perfectly conducting walls.

The slit simulations were performed at an applied bias potential, $\Psi _{bias}$. For the symmetric electrolyte model that we consider in this work, the individual cation and anion chemical potentials are equal in the bulk solution and given by, $\mu _{salt}$. The application of a bias potential, however, generates modified *individual* ion chemical potentials in the pore:

3 $$\begin{aligned} \mu _i = \mu _{salt} - Z_i e\Psi _{bias} \end{aligned}$$

These chemical potentials are used in the grand canonical (GCMC) weights for insertion/deletion moves of the individual ions. This means that the bias potential (the Donnan potential) regulates the excess charge of the slit interior, which is balanced by a surface charge density, $\sigma$. The average surface charge density, $<\sigma>$, is thus an output from a simulation.

When the walls are conducting, we can utilise 3D Ewald summations to handle periodic copies of our simulated system, including image charges [17]. These image charges automatically ensure overall electroneutrality within the pore, as well as a uniformly constant surface potential. With our charge and size symmetric ion model, the bias potential is exactly equal to the so-called Donnan potential (or surface potential), $\Psi$, i.e. $\Psi _{bias} = \Psi$. Narrow pores will necessitate a large number of reciprocal (${{\bar{k}}}$) vectors along *x* and *y* in the Ewald sums, especially since we wish to sample a wide system along these directions, in order to facilitate proper sampling of frozen and fluid phases. In all cases reported (including non-conducting walls) but one, we have set $L = 120$ Å. The exception is the narrowest investigated pore, $h = 5$ Å, with conducting walls. In this case, we settled with $L = 100$ Å, thereby reducing the number of required *k* vectors somewhat.

With non-conducting surfaces, we used a different approach. In this case, the total charge on the surfaces were spread across a large number (400, on each surface) of discrete fractional surface charges. The positions of these wall charges were established by a separate (short) energy-minimizing simulation. GCMC simulations were then conducted with a bias potential (which introduces a charge bias), in a similar manner as for conducting electrodes (eq.(3)). Electroneutrality was ensured by a uniform adjustment of the fractional surface charges. The incorporation of a slab of vacuum between replicas, in the *z* direction, allows us to utilise 3-D Ewald technique. In all cases with non-conducting surfaces, we have used a cubic simulation box (including the vacuum part), with a side length of 120 Å.

The salt chemical potential was adjusted so that the bulk electrolyte is a stable fluid phase, albeit reasonably “close” to a freezing transition. In other words, a relatively modest increase of the chemical potential, or drop of the temperature, will cause the bulk fluid to freeze. This is a relatively common situation for room temperature ionic liquids. With these considerations in mind, we have set $\beta \mu _{salt} = -17.4$ for all GCMC simulations in this work. This results in a stable fluid bulk phase, with a density of about 0.0063 Å$^{-3}$.

## Results

The confined electrolyte may undergo a freezing transition inside the pores. However, the nature of the stable phase depends rather sensitively on the surface separation. In order to establish which phase is stable (fluid or frozen), we have, at each separation, started one simulation from a frozen configuration, and another simulation from a molten configuration. If we (say) find that the initially frozen state melts, we denote this a stable fluid state, and vice versa. There is obviously one caveat: if both phases are at least metastable, we may not observe any phase transition, even after rather lengthy simulations. We will in those cases either include data points for the frozen as well as the fluid state or else by a point “in-between” the two states (see below).

### No applied potential—neutral surfaces

**Fig. 1:**
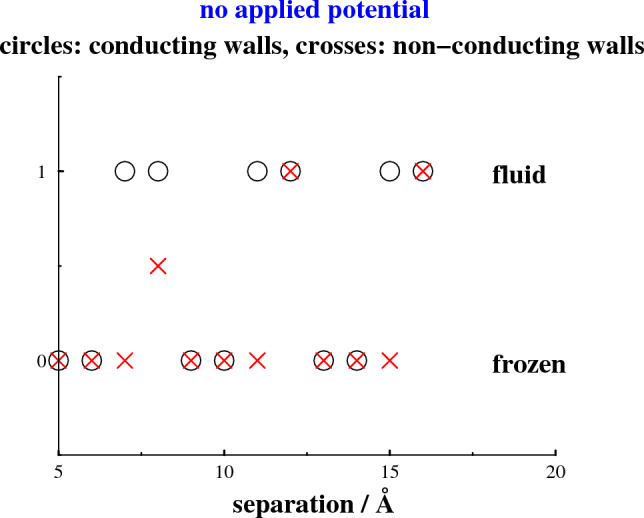
A graph illustrating swaps between stable frozen and fluid pore phases, as the surface separation varies. For illustrative purposes, we have assigned the fluid phase an ordinate (*y*) value of 1, whereas the corresponding assigned value for the frozen phase is zero. Circle symbols denote results obtained from simulations with conducting walls, whereas crosses are for non-conducting walls. A value 0.5 is assigned to a single case, at a separation of 8 Å and non-conducting walls, where we have been unable to determine which of the phases is stable. For all separations above 16 Å, the fluid phase is stable, for conducting as well as non-conducting walls

A pore-induced freezing transition may be observed with neutral surfaces, as the surface separation drops to a few ion diameters. In Fig. 1, we present a separation dependent phase diagram, in the absence of any applied potential, for conducting as well as non-conducting walls. Molten and solid phases are (arbitrarily) assigned a value “1” and “0”. We were not able to determine the stable state at a separation of 8 Å, with non-conducting walls, as both phases are at least metastable and the separating free energy barrier presumably is high. We have indicated this fact by assigning a value “0.5” to this point. With conducting walls, we note an oscillatory pattern, with alternate swaps between the two phases as the surface separation changes. Switching to non-conducting surfaces seems to stabilize the frozen state. This is probably related to a weaker electrostatic screening when the image charges are removed.

**Fig. 2:**
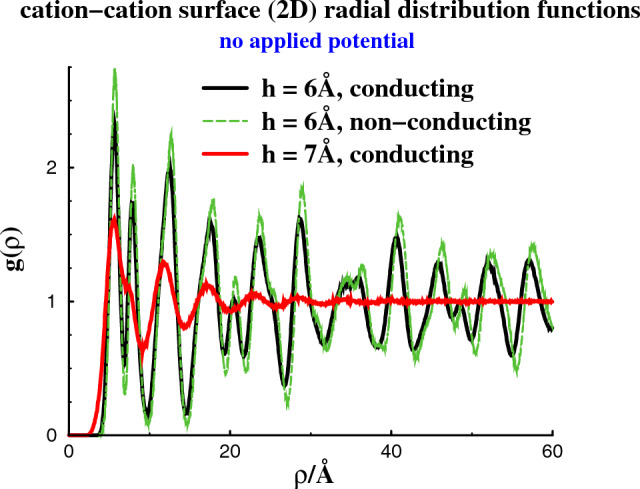
2-dimensional radial distribution functions, $g(\rho )$, where $\rho = \sqrt{x^2+y^2}$. There is no applied potential, i.e. on average the surfaces are neutral. At $h = 6$ Å, the confined electrolyte is frozen, with conducting as well as non-conducting surfaces. Also shown is the distribution function at $h = 7$ Å, with conducting walls. Note that the slits are narrow enough for any *z* dependence to be negligible

Let us now scrutinize the structures of the separate phases. In Fig. 2, we calculate the 2-dimensional radial distribution functions, $g(\rho )$. This distribution is the usual pair correlation function, averaged over the transverse (*z* - coordinate) distances between particles. It can be used to monitor the long-ranged order in the frozen phases that spontaneously form parallel to the surfaces in the absence of any applied potential at a surface separation of 6 Å. This is true for conducting as well as non-conducting surfaces, with the structure being somewhat more pronounced in the latter case. Also shown is how an increased separation between conducting surfaces, to $h = 7$ Å, will melt the confined electrolyte.

**Fig. 3:**
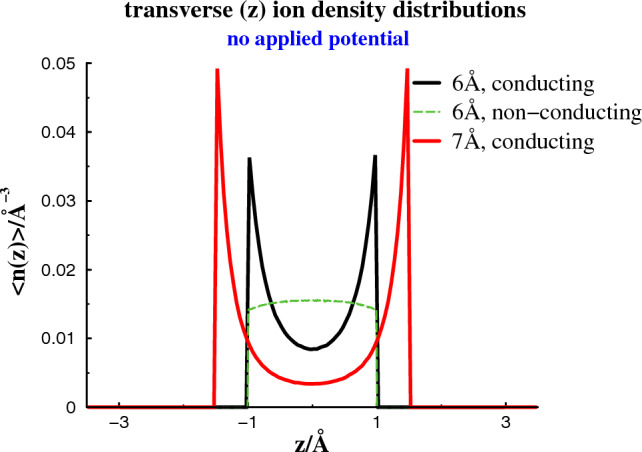
Density distributions along *z*, at a separation of 6 Å, without any applied potential

Corresponding density distributions along the transverse (*z*) direction is provided in Fig. 3, where we note that the phase transition has a less dramatic effect.

In the Supplementary Information, SI, we provide some (*x*, *y*) coordinate snapshots, as a further illustration of the two different phases.

### Finite applied potential

**Fig. 4:**
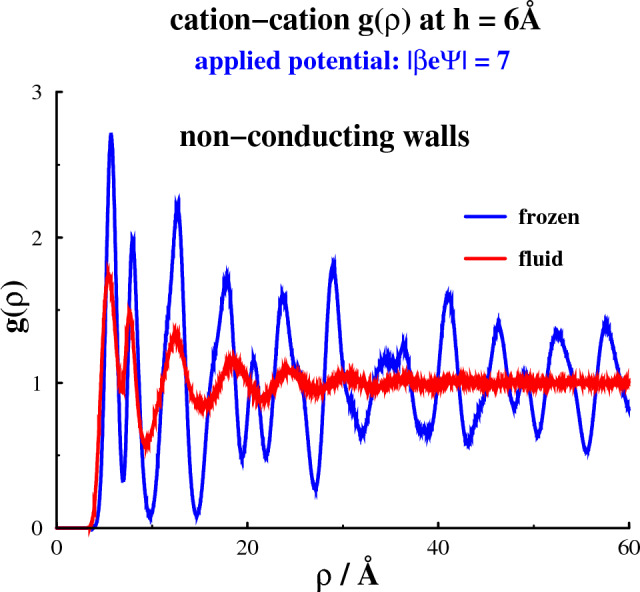
2-dimensional radial distribution functions, $g(\rho )$, with non-conducting walls, separated by 6 Å. The applied (absolute) potential is $|\beta e\Psi | = 7$, in both cases. The blue curve shows data for the frozen state, whereas the red curve is for the fluid phase. One of these phases is likely to be metastable, with the other being fully stable

Applying a potential to a slit containing a frozen electrolyte may result in a first-order phase transition, to a fluid phase. This is probably due to the perturbation that counterions generate to an existing neutral lattice. This may cause the entropic benefits of a fluid phase to dominate, leading to the observed transition. An example of corresponding structural effects is given in Fig. 4, where it is obvious that frozen and fluid phases may coexist at some particular potential. In the displayed case, one of the phases is likely metastable. That is, if we start with initial frozen phase coordinates, for instance obtained at weak or vanishing potentials, then the final state is also frozen. On the other hand, utilising initial fluid phase coordinates (say, from a simulation at high potential), results in a fluid phase.

**Fig. 5:**
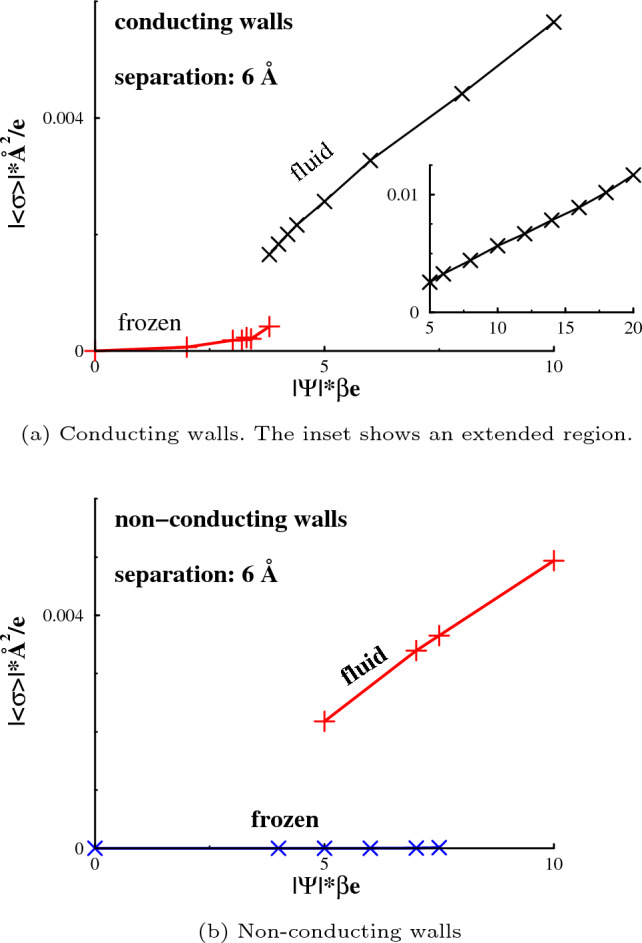
The variation of the average surface charge density with applied potential for systems that are frozen when the surfaces are neutral. The standard deviations of the surface charge density vary with conditions but are always less than $10^{-4}$ e/Å$^2$, and in some cases as low as about $10^{-7}$ e/Å$^2$

In other words, in a pore of a size for which the system is frozen when the walls are neutral, one may induce a first-order phase transition by applying a sufficiently strong potential. This transition is accompanied by a jump in the average surface charge density, as shown in Fig. 5. We also note that a stronger potential is required to generate said transition, when the walls are non-conducting, than when they are conducting. Again, this is most likely related to electrostatic screening effects afforded by image charges. The moderate impact that the phase transitions have on the density distributions along the transverse (*z*) direction, is exemplified in the SI.

The inset of Fig. 5 (a) provides an extension of $<\sigma >(\Psi )$ to strong applied potentials, with conducting walls, where the pore is almost entirely occupied by counterions to the surface charge. For such a narrow pore ($h = 1.5 d$), the superionic state hypothesis suggest a diverging, or at least very rapidly growing, curve under these conditions. This behaviour is, according to the hypothesis, generated by an efficient screening of ion repulsion by image charges. However, our data do not support this, and we find no evidence of a superionic state. The slope of the curve, i.e. the capacitance, is relatively constant from low to high applied voltages, with a fluid phase spanning the region between the surfaces.

The combination of (possible) pore-induced freezing, with near-neutral walls, and pore-induced melting, has substantial implications on the capacitance, $\Delta <\sigma > / \Delta \Psi$, and how the capacitance depends on pore size.

**Fig. 6:**
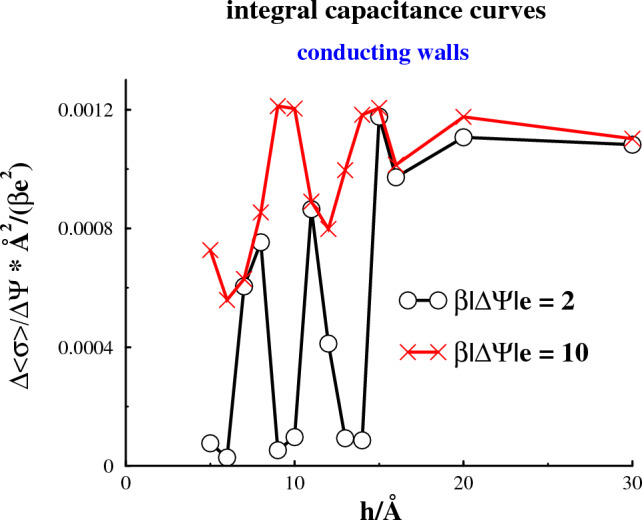
Separation dependence of the integral capacitance, $<\sigma _s>(h)-<\sigma _s^{single}>$, for various applied bias potentials, $\Psi _{bias}$

This is illustrated in Fig. 6, where we see that the integral capacitance curve, as a function of surface separation, depends strongly on the range, $\Delta \Psi$, across which the capacitance is measured. In these cases, we have set neutral walls as our reference, i.e. $\Delta \Psi = \Psi$. A large (absolute) value of $|\Psi |$ will generate a fluid phase, irrespective of which phase is stable at reference conditions (neutral walls). On the other hand, a weak applied $|\Psi |$ may be insufficient to melt an initially frozen phase. A fluid phase has strong ability to respond, via screening, to an applied potential. In a frozen phase, this response is very limited, due to mobility restrictions. This can result in an almost vanishingly small capacitance.

These considerations explain the strongly oscillatory dependence of the capacitance on pore size, for modest values of the applied potential. Oscillations are present also when the capacitance is calculated across a larger potential gap, but then with a considerably smaller amplitude. Another important observation from Fig. 6 is the lack of an “anomalously” increased capacitance for very small pores. As we have already discussed, there are experiments indicating such an increase, but these results are still controversial. Moreover, our results do not lend support to the notion of a “superionic state” in narrow pores with conducting walls. In fact, it would seem that the oscillations we observe are accompanied by an overall *decrease* of the capacitance. However, it should be noted that these findings pertain to fixed electrode area, i.e. an electrode with smaller pores may well display a larger overall capacitance. Finally, it should be mentioned that classical density functional calculations by Jiang et al. [18], on similar systems, were in qualitative agreement with ours, i.e. they found an integral capacitance with an oscillating dependence on pore size.

## Conclusions

This work has highlighted how the relative stability of fluid and solid simple electrolytes in nanoporous environments depend on the degree of confinement, the applied potential, and the polarisability of the confining surfaces. We have established that, with neutral surfaces, the relative stability of the two phases (frozen and fluid) depends sensitively on surface separation, as long as this is narrow. The fluid phase is stable in wide pores, given our choice to use a stable fluid bulk phase. For pore widths where the frozen phase is stable, one may induce a melting transition by applying a surface potential. The system is more able to resist such melting when the surfaces are non-conducting, than when they are conducting. An interesting side effect of these findings, is that the capacitance will be almost vanishing if the applied potential is below the threshold value required to melt the initially frozen electrolyte.

By adopting a symmetric RPM-like ion model, we have excluded mechanisms due to steric asymmetry between the ions. Such asymmetry, as well as geometrical shape, may well be important in many systems, but in this initial work, we have chosen to focus on mechanisms related to Coulombic interactions and ionic screening. Future work will see extensions to more complicated, and more realistic, models.

### Supplementary Information

Below is the link to the electronic supplementary material.

Supplementary file 1 (pdf 167 KB)

## Data Availability

All data generated and analysed in this work are available upon request. This includes all simulation codes.

## Footnotes

1 The phenomenon should not be confused with so-called “superionic conductivity”.
