# Supplementary material for: Phase transitions of ionic fluids in nanoporous electrodes
Source: Eur Phys J E Soft Matter. 2023 Oct 4;46(10):91. doi: 10.1140/epje/s10189-023-00350-2 (PMC10550857; doi:10.1140/epje/s10189-023-00350-2)
Supplement: Supplementary file 1 — (pdf 167 KB) [file 10189_2023_350_MOESM1_ESM.pdf]

Phase transitions of ionic fluids in nanoporous electrodes. Supplementary information

Ayeh Emrani[1], Clifford E. Woodward[2], Jan Forsman[3*]

[1,3]Theoretical Chemistry, Lund University, P.O.Box 124, 221 00 Lund, Sweden..

[2]University College, University of New South Wales (ADFA), Canberra ACT 2600, Australia. E-mail: c.woodward@adfa.edu.au.

*Corresponding author(s). E-mail(s): jan.forsman@teokem.lu.se; Contributing authors: ayeh.emrani@gmail.com; c.woodward@adfa.edu.au;

1 Coordinate snapshots.

In order to further illustrate the nature of the two phases, we will compare (x, y) coordinate snapshots from simulations with non-conducting walls, in a narrow pore ($h = 6$ Å). The applied potential, $\beta\Psi e = 7$, is within the regime where both frozen and molten states can be sampled, depending on the initial condictions, i.e. one of the phases is likely to be metastable. Such snapshots are presented in Figure 1. Note that the overall volume fraction is quite high, and rather similar, in both phases. This is typical for liquid to solid transitions. We will highlight this in a different way, for a system with conducting walls, below.

As a check of our observation that the system may freeze in a narrow pore, we have performed separate simulations, with non-conducting neutral walls, where long-ranged interactions are handled in a totally different manner. Specifically, interactions from ions outside the minimum-image simulation box are approximated by an external potential. This potential is calculated, in a mean-field manner, from previously simulated ion density profiles [1, 2]. Sometimes this approach is referred to as the "charged sheet" metod. An example of typical (x, y) coordinates, resulting from charged sheet simulations in a narrow pore, are provided in Figure 2. The graph clearly demonstrates that the system, which started from randomly distributed ions, ends up in a frozen state.

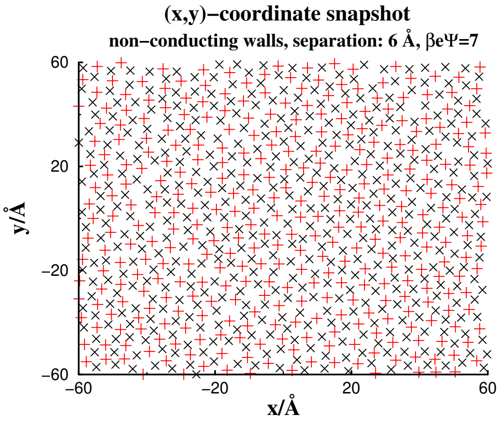

(a) Molten state.

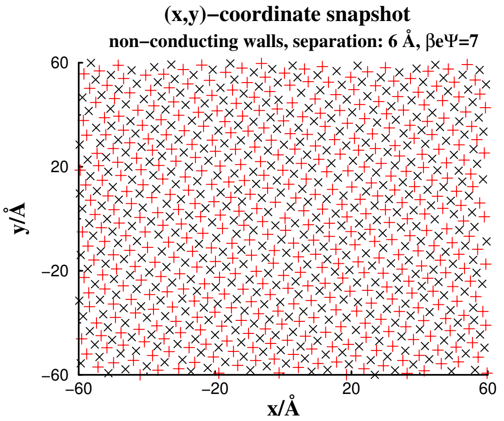

(b) Frozen state.

Fig. 1: Snapshots of (x, y) coordinates, a narrow (h = 6 nÅ) pore with neutral non-conducting walls. The applied potential is of intermediate strength, which allows molten as well as frozen states to be sampled. The end state then depends on the initial state, since both states are at least metastable, and separated by a significant free energy barrier.

2 Ion density distributions.

We have already noted that both the fluid (molten) and frozen states display a high density. Here we will illustrate this in a different manner. With conducting walls, the region of metastability is narrower than when they are non-conducting, possibly resulting from image charge screening effects which might lower free energy barriers.

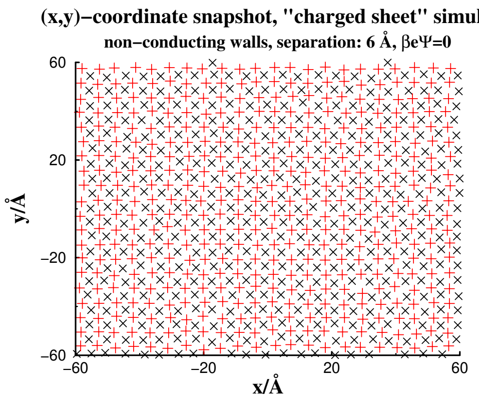

Fig. 2: Final (x, y) coordinates, from "charged sheet" simulations in a narrow ($h = 6$ nÅ) pore with neutral non-conducting walls. These simulations started with a random distribution of ion coordinates.

Nevertheless, at a separation of 6 Å we can establish separate phases, one of which might be metastable, at an applied potential $\beta\Psi e = 3.8$. This can be seen from Figure 5(a) of the main paper.

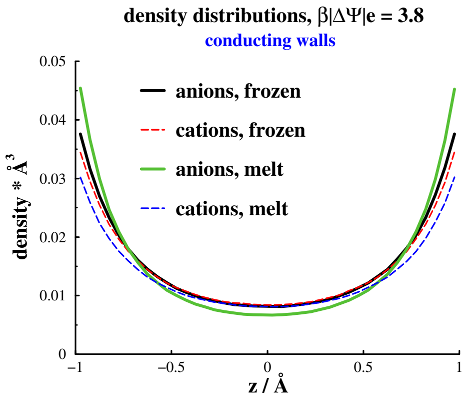

Fig. 3: Density distributions along z, at a separation of 6 Å, and an applied potential $\beta\Psi e = 3.8$. The walls are conducting, and under these conditions it is possible to establish both phases (one of which likely is metastable).

In Figure 3, we see that the ion distributions along the transverse distance (z) are quite similar for the two different phases.

References

[1] Torrie, G.M., Valleau, J.P.: Electrical double layers. i. monte carlo study of a uniformly charged surface. J. Chem. Phys. **73**, 5807–5816 (1980)

[2] Jönsson, B., Wennerström, H., Halle, B. J. Phys. Chem. **84**, 2179 (1980)
